# The Status and Research Progress of Cadmium Pollution in Rice- (*Oryza sativa* L.) and Wheat- (*Triticum aestivum* L.) Cropping Systems in China: A Critical Review

**DOI:** 10.3390/toxics10120794

**Published:** 2022-12-16

**Authors:** Yue Gao, Zengqiang Duan, Lingxiao Zhang, Da Sun, Xun Li

**Affiliations:** 1 State Key Laboratory of Soil and Sustainable Agriculture, Institute of Soil Science, Chinese Academy of Sciences, Nanjing 210008, China; 2 University of Chinese Academy of Sciences, Beijing 100049, China; 3 Technology Extension Station of Agriculture and Fisheries of Nanhu District of Jiaxing, Jiaxing 314051, China

**Keywords:** cadmium pollution status, hazard quotient, average daily intake, pollution mechanism, hotspots

## Abstract

The accumulation of cadmium in rice (*Oryza sativa* L.) and wheat (*Triticum aestivum* L.) is a serious threat to the safe use of farmland and to the health of the human diet that has attracted extensive attention from researchers. In this review, a bibliometric analysis was performed using a VOS viewer (1.6.18, Netherlands) to investigate the status of cadmium contamination in rice and wheat growing systems, human health risks, mechanisms of Cd uptake and transport, and the corresponding research hotspots. It has a certain reference value for the prevention and control of cadmium pollution in rice and wheat planting systems in China and abroad. The results showed that the Cd content in rice and wheat planting systems in the Yangtze River Basin was significantly higher than that in other areas of China, and the Cd content in rice and wheat grains and the hazard quotient (*HQ*) in Hunan Province was the highest. The average Cd concentration exceeded the recommended limit by about 62% for rice and 81% for wheat. The main reasons for the high Cd pollution in rice and wheat growing areas in Hunan are mining activities, phosphate fertilizer application, sewage irrigation, and electronic equipment manufacturing. In this review, we demonstrate that cadmium toxicity reduces the uptake and transport of essential elements in rice and wheat. Cadmium stress seriously affected the growth and morphology of plant roots. In the shoots, Cd toxicity was manifested by a series of physiological injuries, such as decreased photosynthesis, soluble protein, sugar, and antioxidant enzyme activity. Cadmium that accumulates in the shoots is transferred to grains and then passes up the food chain to people and animals. Therefore, methods for reducing cadmium content in grains of rice and wheat are urgently needed, especially in Cd-contaminated soil. Current research on Cd pollution in rice and wheat planting systems focuses on the bioavailability of Cd, soil rhizosphere changes in wheat and rice, and the role of antioxidant enzyme systems in alleviating heavy metal stress in rice and wheat.

## 1. Introduction

Soil pollution and agricultural product safety have become major concerns in China because of increased population growth, industrialization, and limited arable land [1,2]. Humans take up cadmium (Cd) mainly through consuming food, which ultimately affects human health [3,4,5]. Cd has no known biological function in plants or humans but has the highest accumulation rate compared with other heavy metal pollutants [6] and is also a non-essential, highly soluble, non-degradable, and persistent element, mainly occurring in soil [7]. Heavy metal pollution in soils in China is region-dependent, with high levels of pollution reported in the Southern and Eastern regions and low pollution levels observed in the Northern and Western regions [8]. The results from a National Soil Pollution Survey Report [9] conducted from 2005 to 2013 showed that 7.0% of the soil point samples had pollutants of Cd that exceeded China’s recommended limits. Previous findings indicate that 15% of the agricultural soil in the Yangtze River Delta has high heavy metal pollution, especially at the boundary of Zhejiang, Shanghai, and Jiangsu [9]. Xiao et al. [10] (2010) evaluated the characteristics of heavy metal pollution in the soil in the middle and lower reaches of the Yangtze River and reported that the soil was mainly contaminated with Cd, lead (Pb), chromium (Cr), copper (Cu), arsenic (As), and zinc (Zn).

Soil pollution control and the restoration of cultivated land are currently underway in the middle and lower reaches of the Yangtze River to improve soil quality and increase agricultural production output. Cd pollution in rice fields has been widely explored in several parts of the world, including India, Thailand, China, and Japan, and Cd levels in rice pose significant health risks after the consumption of contaminated rice [11,12,13,14,15]. Increased consumption of rice and other grains in the United States has increased Cd intake from dietary sources [16]. Rice and wheat are the most consumed grains by Chinese residents. Therefore, evaluating the content of heavy metals in rice and wheat grains will improve the health of local residents and the national population. Previous studies reported higher Cd contents in wheat and rice than other toxic metals, even when the crops were planted on moderately Cd-contaminated soil [17,18,19,20]. Some soil properties, such as soil pH, soil organic matter (SOM), cation exchange capacity (CEC), and clay content, can significantly influence the mobility and bioavailability of soil Cd and further influence Cd accumulation in crops [21,22]. In addition, some cations in soil solutions (e.g., Mn^4+^, Cu^2+^, Zn^2+^, Si^4+^, and Fe^3+^) may compete with Cd^2+^ for adsorption sites in soil or access to cell walls, cytoplasm, and cell fluids, thereby directly or indirectly affecting Cd accumulation in crops [23,24]. Therefore, in addition to the selection of crop varieties with low Cd accumulation capacity, the regulation of relevant soil properties is an important measure to reduce Cd accumulation in crops.

Notably, the Cd concentrations in grains can exceed recommended Cd thresholds without showing any toxic symptoms in crops [17,19,20]. Therefore, it is necessary to explore the mechanism of heavy metal absorption from soil to crops and the key regulatory factors of heavy metal absorption by different food crops. Rice plants tend to accumulate more Cd than other grains [25,26,27,28]. Identifying key transporters and their role in Cd accumulation or detoxification will provide useful information for the development of biological breeding to reduce Cd levels in rice grains [29]. The key transporters of Cd uptake by rice roots have been reported to be similar to the transporters of essential elements such as zinc, manganese (Mn), and iron (Fe) [30]. Metal chelators and several organic acids also play an important role in reducing the toxicity of Cd to essential organelles and macromolecules. Some key detoxification genes and related mechanisms have been explored [31,32]. However, it is not clear whether plant hormones are involved in Cd detoxification or tolerance. Due to physiological differences, crop varieties are important factors affecting Cd accumulation ability [33]. Selection of rice varieties with low Cd accumulation and irrigation management may also be effective strategies for reducing Cd in rice, but these options still need further development [34]. Wheat-derived products are the main source of human ingestion of Cd. Cd is more toxic to wheat than other toxic metals, such as chromium [35]. Cd toxicity reduces the uptake and transport of essential elements by plants, including wheat [36]. Under Cd stress, wheat root growth and morphology were seriously affected. In shoots, Cd toxicity presents a number of physiological impairments, such as decreased photosynthesis, soluble protein and sugar, and antioxidant enzyme activities [37,38,39]. Cd that accumulates in the shoots can be transferred to grains and then through the food chain to people and animals. Therefore, reducing Cd content in wheat is an important requirement, especially in Cd-contaminated soils. Different mitigation strategies have been used to reduce Cd toxicity in wheat. These strategies may include plant growth regulators (PGRs), appropriate application of mineral nutrients, silicon, inorganic modifiers, biochar, manure, and compost [40,41,42,43,44]. Agronomic management practices, such as wheat varieties with low Cd accumulation, crop rotations, planting patterns, and the application of microorganisms, can also be used to reduce Cd uptake and toxicity in wheat [45,46,47].

Rice and wheat are major crops and staple foods in China. In order to better understand the Cd pollution in rice and wheat planting systems in China, the crops’ main production areas in eleven provinces in the Yangtze River Basin and the southeastern Yellow River Basin were taken as examples. Accordingly, the specific objectives of this study were to (i) summarize the current situation of Cd pollution in rice and wheat growing areas in China from 2000 to 2022 by collecting the data from the previous literature in order to understand the distribution of Cd pollution, (ii) assess the human health risks of Cd through contamination status and intake, aiming to highlight the areas with high human health risk to which more attention needs to be paid in the future, (iii) review the mechanisms and influencing factors of Cd uptake and transport in rice and wheat, aiming to find out the methods for reducing the Cd content in grain, and (iv) summarize the research hotspots and dominant research institution through bibliometric analysis in order to look forward to the development trend in this research area.

## 2. Materials and Methods

### 2.1. Literature Search

Articles published in English and Chinese from January 2000 to June 2022 were reviewed in this study. Articles that reported Cd pollution status, health risks, Cd pollution mechanism, and research hotspots in the rice and wheat cropping system in China were included in this review. The search was conducted using the Web of Science (WOS), the Science Direct, and the China National Knowledge Infrastructure Engineering database (CNKI). The search method used “topic.” Keywords such as “Rice and wheat cropping system”, “Wheat/rice cadmium”, “Cadmium pollution in wheat or rice”, “Wheat enriched with cadmium”, “Rice enriched with cadmium”, “Wheat cadmium”, “Rice cadmium”, “Wheat/rice rotation cadmium”, “Remediation of cadmium pollution in rice”, “Remediation of cadmium pollution in wheat”, “Wheat cadmium water and fertilizer management”, “Rice cadmium water and fertilizer management”, “Low accumulation varieties of rice cadmium”, “Low accumulation varieties of wheat cadmium”, “Rice cadmium remediation materials”, and “Wheat cadmium remediation material” were used to search for relevant papers in this review.

### 2.2. Data Retrieval and Analysis

Data retrieved from each article included: (i) the name of the first author, title, publication source, country of issue, and publishing year; and (ii) the location of the study area. The location of the study area was inferred from the scope of the administrative division if it was not clearly stated in the article. Included articles were reviewed to ensure that they met the following criteria: (i) the cropping method of farmland reported in the study was the rice and wheat cropping system (not only the rice-wheat rotation but also growing rice and wheat in the same place); (ii) sources of pollution in the study area were mainly from human activities, including industrial waste gas, waste water and waste residue, mining development, and agricultural waste; and (iii) accurate analysis and presentation of data, including mean value, deviation, and coefficient of variation was reported in the articles. This review was conducted in May 2022 by reviewing literature and retrieving relevant data from previous studies. A total of 10,465 articles that met the study criteria were obtained from the search process. The VOS viewer (1.6.18, Netherlands) software was used to perform a bibliometric analysis of the search results from the aspects of Cd accumulation in agricultural land, research hotspots, and future prospects. A spatial distribution map of Cd pollution in rice and wheat cropping system was generated using Arc Gis (10.3) software.

### 2.3. Risk Assessment

The Hazard Quotient (*HQ*) proposed by the US Environmental Protection Agency (EPA) was used to assess the non-carcinogenic human health risks of exposure to hazardous substances. The non-carcinogenic risk of Cd was expressed as the *HQ*, as shown below:

(1) $$HQ=\frac{ADI}{RfD}$$

where *ADI* represents the average daily intake of Cd (μg kg^−1^ *BW* day^−1^), and *RfD* denotes the chronic reference dose of Cd. The oral reference dose of Cd was 1 μg kg^−1^ *BW* day^−1^ [48]. An *HQ* value ≤ 1 meant that significant adverse reactions were unlikely to occur in the exposed population. An *HQ* value > 1 showed a high non-carcinogenic risk to the exposed population. The *ADI* of Cd was calculated using the following equation:

(2) $$ADI=\sum \frac{C_{i}\times IR_{i}}{BW}$$

where *ADI* denotes the estimated daily intake of Cd (μg kg^−1^ *BW* day^−1^); $C_{i}$ represents the concentration of Cd in rice, leafy, rootstalk, and legume vegetables (mg kg^−1^); $IR_{i}$ represents the daily consumed amount of rice, leafy, rootstalk, and legume vegetables (g day^−1^); BW indicates the average body weight of the corresponding population (kg body weight, kg *BW*). The $C_{i}$ of rice was converted using a factor of 0.86 × 0.70 because rice stored at home usually contains 14% water content [49], and during brown rice processing (milling), the content of Cd in polished white rice is reduced by 20~40% (mean: 30%) [50,51]. The average monthly intake (*AMI*) of Cd was calculated using the following equation:

(3) AMI=ADI×30

The possible exposure to Cd from rice and vegetables in Xiangtan County was investigated. Potential health risks associated with dietary Cd intake were estimated through Monte Carlo simulations [52,53], and 30,000 iterations were performed using the Crystal Ball 11.1 tool to obtain reliable results. Data on consumption rate ($IR_{i}$) and body weight (*BW*) were retrieved from the Chinese National Nutrition and Health Survey (CNNHS) conducted in 2002 [54]. As a major nutritional reference database for the Chinese population, the CNNHS contains the dietary patterns of 67,608 people in 31 provinces, autonomous regions, and municipalities across China.

## 3. Cd Pollution in Rice and Wheat Cropping System

### 3.1. Cd Concentrations in Rice and Wheat Grain

**Figure 1:**
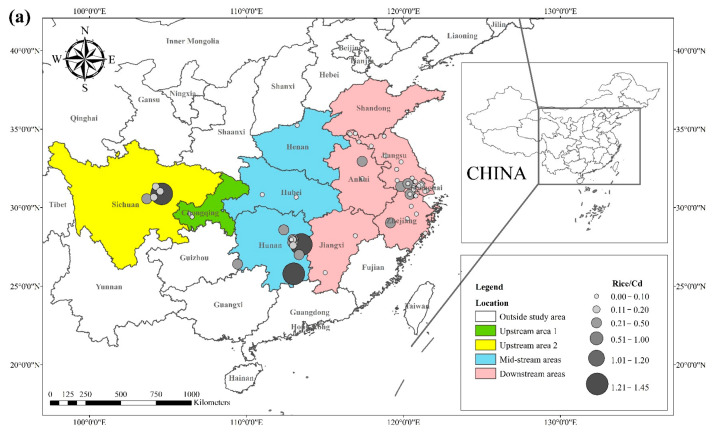

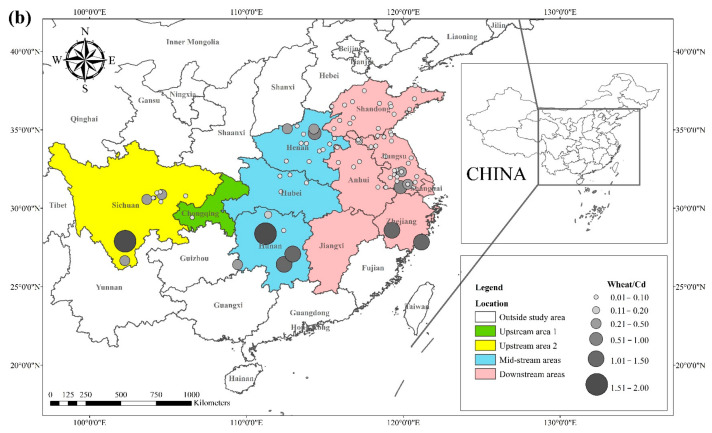
Distribution of Cd concentrations in (**a**) rice and (**b**) wheat, based on data collected from previous studies.

The literature survey results showed that the regions where both rice and wheat were grown were mainly in nine provinces and two municipalities around the Yangtze River Basin and southeast of the Yellow River Basin, including Sichuan, Chongqing, Hubei, Hunan, Jiangxi, Anhui, Zhejiang, Jiangsu, Shanghai, Shandong, and Henan provinces. Table 1 shows the results of other studies on the Cd pollution of rice and wheat cropping systems in China. The retrieved data showed that the average concentration of Cd in rice ranged from 0.0070 mg kg^−1^ to 1.45 mg kg^−1^. The proportion of studies where the Cd concentration in rice was lower than the national food safety standard limit (GB 2762-2017) (0.2 mg kg^−1^) was 79%. The spatial distribution of Cd concentration in rice is presented in Figure 1a, with larger dots representing higher Cd concentrations. Studies on excess Cd content in rice were mainly conducted in the central part of Sichuan province in the upper reaches of the Yangtze River, the southwest of Chongqing, the southeast and northeast of Hunan, as well as the northern part of Anhui, southern Jiangsu, and northern Zhejiang in the Yangtze River Delta region. Studies on Cd accumulation in rice have also been conducted in other major rice-producing countries, including Thailand, Bangladesh, Indonesia, and India. Effective methods should be formulated to remediate Cd-contaminated rice in Punjab (India, 0.99 mg kg^−1^) and Mae Tao (Thailand, 0.329 mg kg^−1^), where the average Cd concentration in rice exceeds the required limit. The spatial distribution of Cd concentrations in wheat is shown in Figure 1b. The average concentration of Cd in wheat ranged between 0.0080 mg kg^−1^ and 2.0 mg kg^−1^, and the proportion of studies where the Cd concentration in wheat was lower than the national food safety standard (0.1 mg kg^−1^) was 83%. Studies on excessive Cd concentration in wheat were mainly conducted in the southern part of Sichuan in the upper reaches of the Yangtze River, the southeastern and northern parts of Hunan Province, the southern part of Jiangsu, and the southern part of Zhejiang in the Yangtze River Delta region, as well as Shandong and Henan provinces in the southeast of the Yellow River Basin. The Cd pollution of wheat in the rice and wheat cropping system was relatively low compared with the Cd pollution in rice, and the pollution area was smaller for wheat.

The Cd content in rice and wheat in Hunan Province increased significantly. Studies reported that the average concentration of Cd in rice and wheat was 0.21 mg kg^−1^ and 1.08 mg kg^−1^, respectively. Hunan province is known for its rich non-ferrous metals and extensive mining industry. The elevated Cd concentration in the paddy soils in Hunan province was highly correlated with the mining activities, and non-ferrous metal smelting was mainly conducted in the Xiangjiang River Basin. Studies on Cd pollution in paddy fields in the Xiangjiang River Basin showed that the average concentrations of Cd in paddy soils in Chenzhou, Xiangtan, Hengyang, Zhuzhou, Changsha, and Xiangyin city were 3.01, 1.93, 1.73, 0.50, 0.39, and 0.66 mg kg^−1^, respectively, all exceeding the recommended level [95,96].

### 3.2. Cd Hazard Quotients in Rice and Wheat

**Figure 2:**
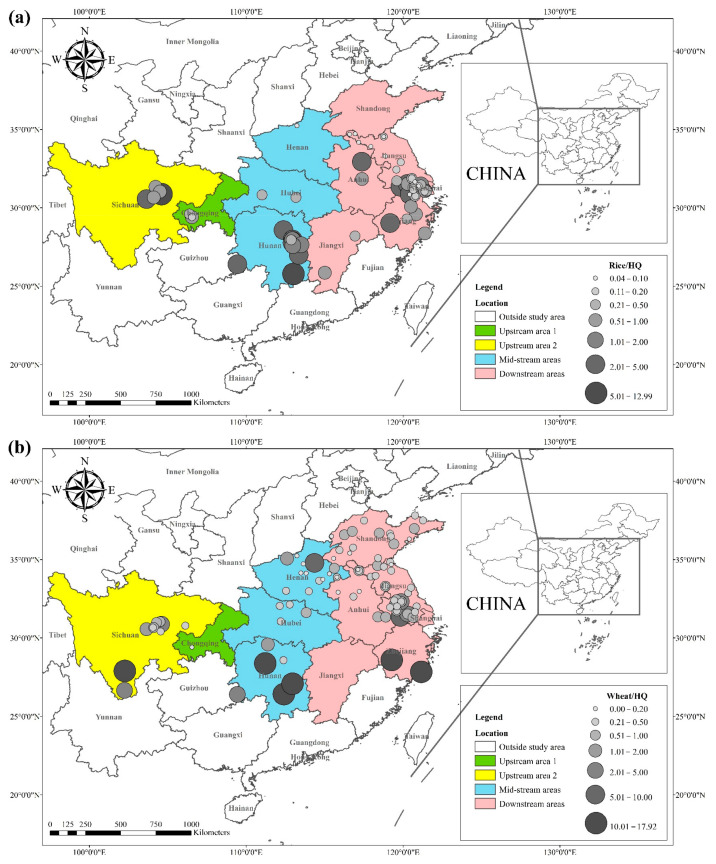
*HQ* of Cd in (**a**) rice and (**b**) wheat, based on data collected from previous studies.

The health risks of exposure to heavy metals through oral, dermal, and inhalation routes can be assessed using a health exposure risk equation. The risk of exposure through dietary Cd intake is expressed in terms of *ADI* and *HQ*. The World Health Organization (WHO) has established a provisional tolerable daily intake (*PTDI*) for Cd of 0.06 mg kg^−1^ day^−1^; exposed populations have high health risks when the *HQ* is greater than 1. Dietary Cd intake poses a threat to local populations due to the combined effect of high grain intake rates and high grain Cd concentrations. The overall Cd concentration in Hunan province is significantly higher than that of other regions due to the extensive industrial activities in this region. The average Cd concentration in rice in Hunan exceeds the recommended limit by approximately 62%, and the average Cd concentration in wheat exceeds the recommended limit by about 81%. The spatial distribution of dietary Cd intake (*HQ*) in rice and wheat is shown in Figure 2, with a larger point indicating a higher risk factor. Findings from reviewed studies showed that the average risk factor for Cd in rice was 1.20, and the average risk factor for Cd in wheat was 1.54. The areas with a high *HQ* of Cd in rice were mainly located in the Yangtze River Basin and the southeastern coastal areas, including Sichuan, Hunan, Anhui, Jiangsu, and Zhejiang. Studies conducted in Chongqing, Hubei, and Shandong showed that the risk factor for Cd was low in these regions. The areas with a high *HQ* of Cd in wheat were primarily located in Sichuan, Hunan, Anhui, Jiangsu, Zhejiang, and Henan, and the risk factors for Cd in Chongqing, Hubei, and Shandong were lower compared with other regions. Consumption of locally produced rice and wheat increased the intake of Cd by the local population.

**Figure 3:**
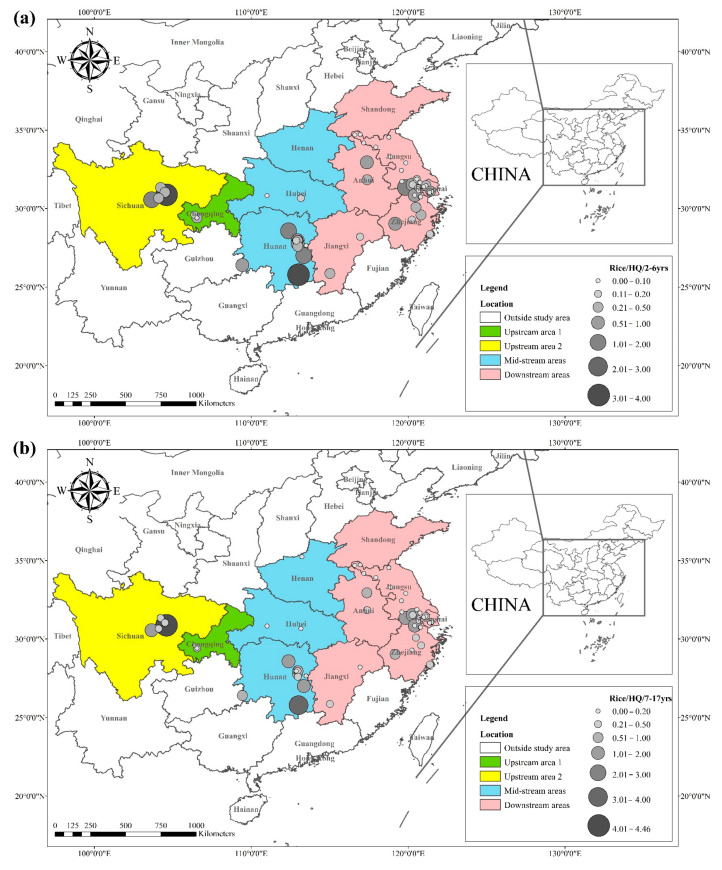

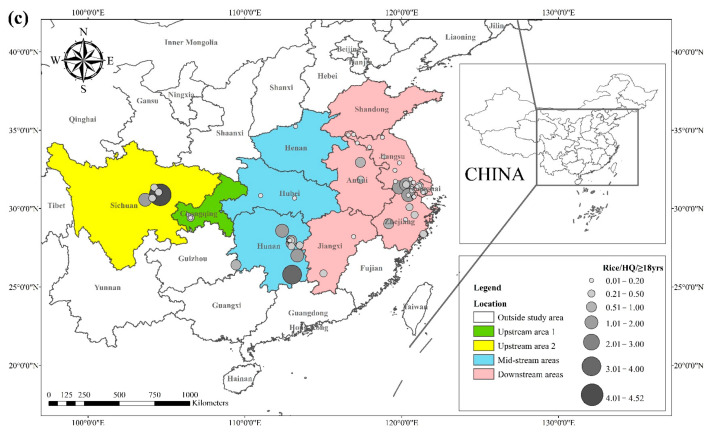
(**a**) *HQ*/2–6 yrs., (**b**) *HQ*/7–17 yrs., and (**c**) *HQ*/ ≥ 18 yrs. of Cd in rice, based on data collected from previous studies.

**Figure 4:**
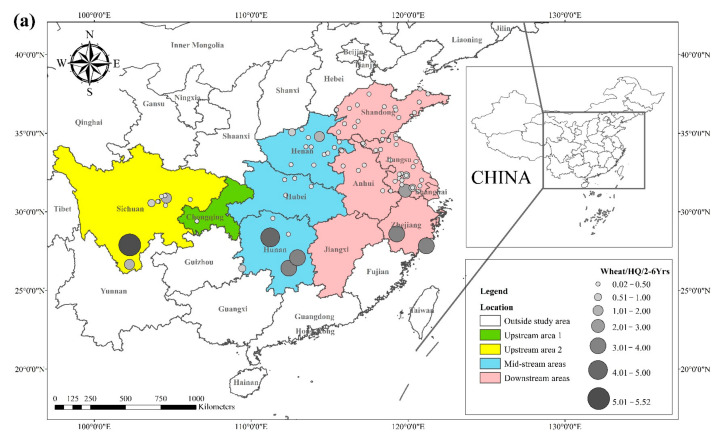

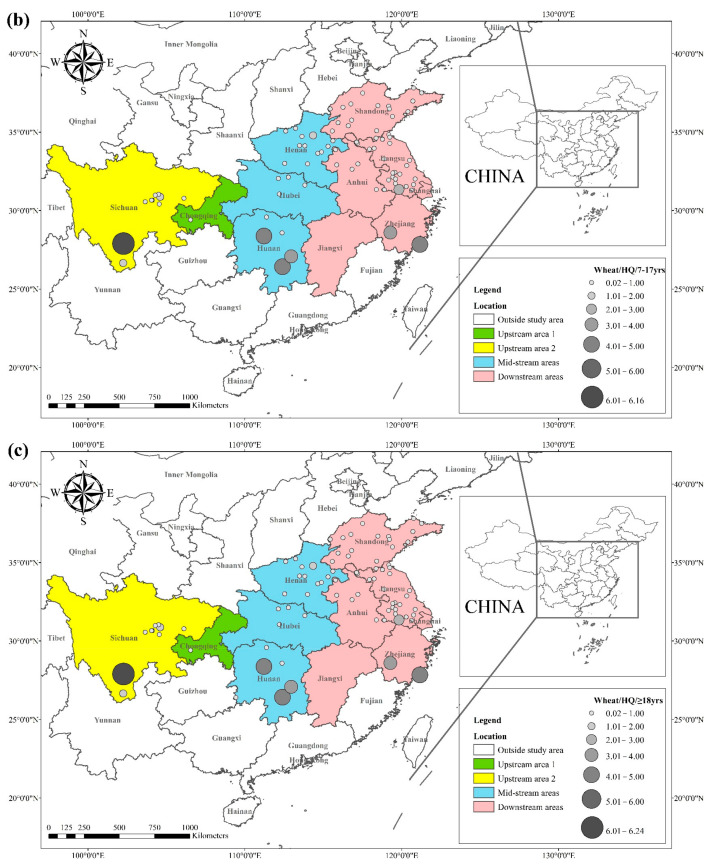
(**a**) *HQ*/2–6 yrs., (**b**) *HQ*/7–17 yrs., and (**c**) *HQ*/ ≥ 18 yrs. of Cd in wheat based on data collected from previous studies.

The *HQ* for the dietary consumption of rice and wheat for the corresponding populations (children (2–6 yrs.), adolescents (7–17 yrs.), and adults (≥18 yrs.)) are presented in Figure 3 and Figure 4. The areas with a high *HQ* dietary intake of Cd in rice for children, adolescents, and adults were mainly located in central Sichuan, central and eastern Hunan, northern Anhui, and southern Jiangsu. The *HQ* value for rice and wheat in Hunan province was 1.8 and 2.6 times higher than the national average dietary Cd intake of 15.3 μg kg^−1^ *BW* month^−1^, respectively, according to the 2015 General Diet Study [38,97]. In contrast, dietary Cd exposures in populations outside Asia were generally very low. A previous study reported that the Cd exposure in the European population was 6.4~9.6 μg kg^−1^ *BW* month^−1^ [98], whereas the Cd in Australia was 2.2~6.9 μg kg^−1^ *BW* month^−1^, and that in the United States was reported as 4.6 μg kg^−1^ *BW* month^−1^ [99]. The areas with a high *HQ* of dietary Cd in wheat for children, adolescents, and adults were primarily located in southern Sichuan, central and eastern Hunan, southern Jiangsu, and southern Zhejiang, with *HQ* values greater than 1. Notably, Sichuan and Hunan provinces exhibited average dietary Cd intakes 5.5 and 8.1 times higher than the recommended intake, respectively.

### 3.3. Cd in Rice-Wheat Cropping Field

A high *HQ* is positively correlated with a high concentration of Cd in soil. The first step in preventing Cd in grain is to identify and block major sources of contamination in the soil-based rice/wheat system. A high Cd concentration in agricultural soils mainly results from the geological background derived from the parent material and from human activities, such as the deposition of the atmosphere, mining activities, irrigation using wastewater, the application of phosphate fertilizers, livestock manure, organic fertilizer, and sludge application. Cd is a naturally occurring non-essential trace element, and the average concentration of Cd in soils is 0.01~2.00 mg kg^−1^ globally, whereas the average concentration in China ranges from 0.10~1.80 mg kg^−1^ [100]. Cd mainly exists as a complex with sulfur. Cd is released from this complex into the surface environment during mineral rock weathering. Parent materials determine the Cd content in soil systems. Soils derived from parent materials inherit the characteristics of the parent materials and their trace element content [101]. A study conducted in the Khorat Basin of Thailand showed that the different properties of parent materials significantly contributed to the chemical composition of paddy soils [102]. In addition, a study in Guizhou, China, reported that dry land topsoil derived from carbonate rocks had higher levels of Cr, Cd, and Hg [103]. The soil background value represents the contribution of the parent material, which exhibits a complex and diverse distribution in China. Geographic differences in soil Cd background values are observed, with higher background values reported in some provinces, such as Guizhou and Guangxi. Although there was no notable anthropogenic source of heavy metal pollution in the sampling area, an elevated Cd content was observed in paddy soil samples from the Cd geological anomaly areas in Guangxi and southwest China regions, which can be attributed to the weathering of carbonate rocks [104,105,106,107].

Human activities significantly affect the accumulation of heavy metals in farmland ecosystems. Mineral resources are indispensable materials for industrialization. The output and consumption of China’s mineral resources account for 19% and 35% of the global rates, respectively [108]. Heavy metals and acid mine wastewater (AMD) produced in the process of mining are the main sources of pollution that pose a potential health threat to the environment. Acidic water containing heavy metals penetrates into the bottom layer of paddy soil through the pores of waste rock piles and tailings ponds, eventually causing toxicity to organisms [109]. In the Dabaoshan mining area, AMD with high levels of Cd (0.1 mg L^−1^) is directly discharged into the Hengshi River, and irrigation using the river water results in an approximately 5.5 mg kg^−1^ Cd concentration in the downstream paddy fields [110,111]. Most Cd and non-ferrous metal deposits are located in major rice-producing areas in China. Several mining activities in Yunnan, Guizhou, Hunan, and Jiangxi are located around the Yangtze River Basin. Illegal civilian mining activities have significantly increased since the early 1980s, mainly in Hunan, Guangdong, and Guizhou regions [112]. These unregulated mining activities cause severe heavy metal pollution in the surrounding environment. The atmospheric deposition of particulate matter (PM) from mining and smelting also contributes to increased Cd concentrations in paddy soils. In addition, the rapid development of China’s agriculture has resulted in increased utilization of agrochemicals, such as fertilizers. China has been a major consumer of global fertilizers since 1994. Fertilizers are major sources of heavy metal pollution in arable land, especially phosphate fertilizers, which significantly contribute to Cd pollution [113,114,115,116]. The raw materials for the production of phosphate fertilizers include phosphate rock, which contains varying concentrations of Cd. A previous study reported that the average Cd concentration in 159 phosphate fertilizer samples in China was 0.77 ± 2.42 mg kg^−1^. The Cd content in phosphate fertilizers in China was significantly lower than that in European countries (mean: 7.40 mg kg^−1^) [117,118]. The application of mineral and organic fertilizers accounts for ~63% of the total annual Cd input into agricultural land [119]. The electronics industry is also the main source of Cd pollution in paddy soil. Illegal recycling and extensive disposal of e-waste result in Cd pollution in farmland soils in China, Nigeria, and India [120,121]. Other sources of Cd in agricultural soils include intensive livestock production, urbanization, animal manure, and sewage sludge [122,123,124,125].

## 4. Mechanism of Cd Pollution in Rice and Wheat Cropping System

### 4.1. Transport of Cd in Rice and Wheat Cropping System

The negative effects of Cd on crop physiological and biochemical processes, such as protein metabolism, mineral absorption, and photosynthesis, have been widely studied in the past [126,127]. The excessive accumulation of Cd in crops will significantly affect the growth and development of crops and lead to the further accumulation of Cd. Cd accumulation in crops is, therefore, influenced by a variety of soil factors, as well as the bioavailability and environmental availability of the crop to Cd. Environmental availability represents the content of heavy metals in soil, mainly including the proportion dissolved in pore water and the proportion adsorbed on the surface of soil minerals. Bioavailability refers to the portion of heavy metals dissolved in pore water that can be absorbed by the plant, while toxic bioavailability refers to the portion that accumulates in the plant and may be toxic to the plant [128,129].

The transport of bioavailable Cd from soil to rice grains is via coplastids that absorb roots and via root cells that load or sequester xylem. Cd is then transported through the xylem to the shoots and through the xylem to the nodes. The transport and accumulation of phloem to grain begins. Cd is a transcellular process regulated by a variety of transporters, such as absorption by root isoplasts, loading or isolation by root cells in the xylem, transport to nodes through the xylem, and transport to grains through the phloem. For example, *OsIRT1* (IRT, iron-regulated transporter) and *OsNramp1* (Nramp, natural resistance-associated macrophage protein) transporters are involved in the uptake of Cd in roots. The *OsHMA3* protein regulates Cd sequestration in root cell vacuoles, whereas *OsHMA2* (HMA, a P1B-type ATPase) regulates Cd delivery to developing tissues. *OsLCT1* (*OsLCT1*, a low-affinity cation transporter) is a transporter associated with phloem Cd transport [106]. In the transport of Cd from soil to grain, xylem loading and transport is the first rate-controlling step, while phloem transport and unloading is the last rate-limiting step. Roots and nodes are the basic barriers to Cd transport to brown rice grains [130,131].

Cd uptake in wheat plants is mainly through the roots [132,133]. The rate of Cd uptake by wheat varies with soil type, air pollution, and wheat variety [134,135,136]. In addition, root exudates play an essential role in the accumulation of Cd in wheat [136]. A recent study showed that chlorine (Cl^−^ could activate Cd in soil and increase the rate of Cd uptake by wheat. Depending on the wheat variety, the high and low concentrations of Cd accumulated by the root system after Cd uptake are transferred to the shoots [137,138]. Studies report that the higher retention of Cd in roots can be attributed to the chelation of Cd by organic acids [132]. However, another study reported that phytochelation might not be a limiting factor for the differential storage of Cd in wheat roots [139]. Shoot Cd accumulation in wheat depends on root-cadmium transport, whereas Cd accumulation in wheat grain mainly depends on root-shoot Cd transfer and the indirect transport of Cd from root to grain through xylem-to-phloem of the stem [140,141]. Riesen and Feller (2005) reported that Cd could be reactivated in wheat by the phloem and transpiration process [142]. Rice exhibits significant differences in Cd transport and accumulation among various genotypes. A study was conducted on 38 rice genotypes (indica vs. japonica), and the results showed that Cd concentrations ranged from 0.06 mg kg^−1^ to 0.99 mg kg^−1^, with significantly higher Cd accumulation levels in indica rice than in japonica rice [143]. Differences in grain Cd accumulation between different genotypes can be attributed to the differences in the transport of Cd from vegetative organs such as leaves and stems to the reproductive parts [144]. Cd transport in the rice-soil ecosystem is regulated by various transporters. Therefore, the expression level of the transporter gene and the content of the transporter affect the level of accumulation of Cd in rice. Overexpression of the *OsHMA3* protein limits Cd accumulation in rice grains and enhances Cd tolerance [145]. In addition, the co-expression of *OsHMA2*, *OsLCT1*, and *OsZIP3* effectively inhibits Cd transfer and accumulation in rice grains [146].

For safe wheat production, people have tried to screen and plant low-Cd-accumulating wheat varieties in light and moderate Cd-contaminated farmland [147,148]. Zhang et al. planted 8 wheat varieties in Cd-contaminated soil with a soil Cd content of 1.12 mg kg^−1^ (pH = 7.15) [148], of which 6 wheat varieties had a grain Cd content lower than the national food safety standard (0.10 mg kg^−1^). Chen et al. (2017) screened out 10 low-Cd accumulation varieties from 261 wheat varieties and planted them in Cd-contaminated farmland with a soil Cd content of 2.4 mg kg^−1^ (pH = 7.35) [149]. Low Cd accumulation in wheat cultivars is related not only to its genetic characteristics, such as low expression of transporters [150], smaller root morphological parameters (root length, surface area, and volume), etc. but also to its rhizosphere exudates and microbial communities [151]. Variety is an important factor affecting the Cd content of wheat grains. In some densely populated countries such as China and India, the use of heavy metal-contaminated farmland for agricultural production is inevitable, and planting crops with a low accumulation of heavy metals is a cost-effective measure to achieve food security in these regions.

### 4.2. Factors Affecting Transport of Cd in Soil

A prediction model of rice/wheat grain Cd concentration based on soil properties and Cd concentration in soil is presented in Table 2. This model showed that in addition to soil pH, soil Cd content and SOM were important factors regulating the accumulation of Cd in rice grains. In the actual farmland ecosystem, there is no quantitative relationship between the heavy metal content in soil and that in crops. Crops grown in soils with high concentrations of heavy metals do not necessarily have high concentrations of heavy metals [152]. The accumulation of heavy metals in crops is correlated with crop varieties and soil physicochemical properties. Soil physicochemical properties, such as soil pH, soil organic matter (SOM), cation exchange capacity (CEC), clay content, and soil aggregate structure, affect the Cd accumulation in crop grains. Notably, soil pH is the most important factor in determining the level of accumulation of Cd in grains. The adsorption of Cd in the soil is highly dependent on pH, and the percentage of the exchangeable Cd component increases with the decrease in soil pH [153,154,155]. The study of Kicińska et al., (2022) showed that the pH of Cd-contaminated soil decreased by 3.5~3.7 units, and the Cd content in the soil solution increased by 2.00 mg kg^−1^ [156]. Mao et al., (2019) observed that low pH promotes heavy metal pollution in paddy soils in the Yangtze River Delta [157]. The results showed that rice accumulated more Cd under low pH conditions. Excessive utilization of nitrogen fertilizers resulted in severe acidification of paddy soils in southern China. A previous study reported a decrease in pH from 6.64 in 1988 to 6.05 in 2012 [158].

A high SOM content in the soil is correlated with a low Cd content in ionic and complex forms, and a high SOM content reduces the bioavailability of Cd [159]. Soil SOM and Cd form macromolecular complexes or precipitated compounds, which are not easily absorbed by crops [160]. Chen et al. found through UV-visible/fluorescence spectroscopy that the main oxygen-containing functional carboxyl groups and phenolic hydroxyl groups of organic matter complexed with Cd to form humus-Cd complexes, which reduced the availability of soil Cd [161]. Li et al. (2022) found that the application of phosphorus fertilizer significantly reduced the content of available Cd in the soil [162], which was attributed to (i) the application of phosphorus fertilizer increasing the negative charge and soil pH, which in turn enhanced the adsorption of Cd by soil particles; and (ii) the increase in available phosphorus promoting the formation of Cd precipitates such as Cd_3_(PO_4_)_2_ and Cd_5_(PO_4_)_3_OH/Cl in the soil. A previous study reported that these nutrients competed with Cd for various transporters on the cell membrane [163]. In addition, SOM improves the vigor of rice and its ability to absorb nutrients, such as copper and zinc, thereby enhancing the resistance of rice crops to Cd [164].

Several studies have been conducted to explore the relationships among soil microbes, crops, and heavy metals. Researchers report that microorganisms alter the bioavailability of heavy metals in soil through various mechanisms, such as biosorption, crop cell accumulation, precipitation, and redox reactions [165,166,167,168,169]. Microorganisms have functional groups such as carboxyl (-COOH), amino (-NH_2_), and hydroxyl (-OH), which can bind to Cd^2+^ and remove it through cell wall surface solutions [135,170,171]. Intracellular bioaccumulation and intracellular lysis of Cd-tolerant bacteria reduce Cd bioavailability [172]. The previous findings indicated that Cd accumulation in rice plants was reduced after inoculation of Cd-tolerant bacteria that converted Cd^2+^ to precipitated Cd, ultimately reducing Cd bioavailability.

**Table 2:** The prediction model for Cd content in rice or wheat grains.

Model	Grain	R^2^	*p*	Reference
Y_DTPA-Cd_ = 0.152 + 0.05X_soil total Cd_ −0.029X_soil pH_ + 0.006X_SOM_	rice/wheat	0.53	<0.01	[173]
LogCd_wg_ = 0.703 + 1.041LgCd_soil_ − 0.175pH	wheat	0.783	<0.01	[174]
logCd_wg_ = − 0.383 + 0.824logeCd	wheat	0.375	0.01	[175]
LogCd_rg_ = 1.38 + 0.41LogCd_soil_ − 0.183pH − 0.09SOM	rice	0.51	<0.001	[155]
Log(Cd_rg_) = − 0.369 − 0.068pH + 0.153logCd_soil_	rice	0.565	<0.0001	[176]
Log(10^3^ Cd_rg_) = 3.301 + 0.534lgCd_soil_ − 0.211pH − 0.012SOM + 0.006CEC	rice	0.448	0.000	[177]

DTPA-Cd and eCd are the concentrations of available and total Cd contents in soil, respectively; Cd_rg_ and Cd_wg_ are the concentrations of Cd in rice and wheat grain, respectively.

## 5. Research Progress and Key Study Areas

### 5.1. Analysis of Publication Sources, Discipline Categories, and Annual Trends

**Figure 5:**
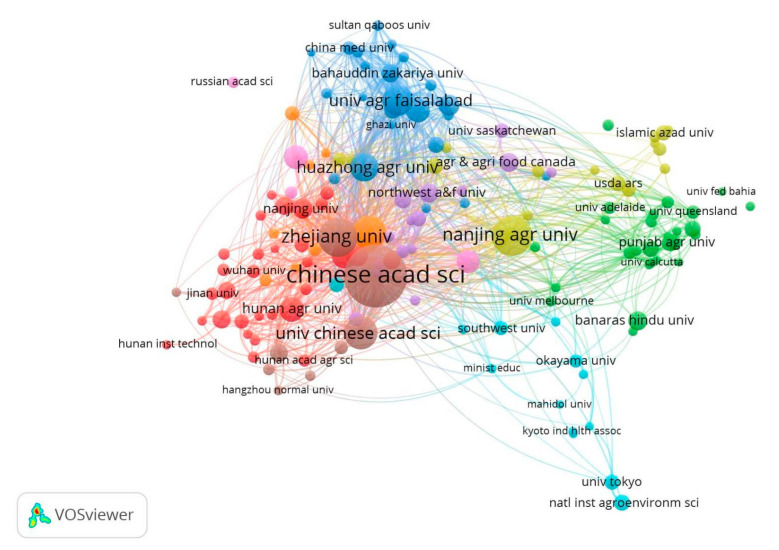
Analysis of research cooperation relationships between institutions.

**Figure 6:**
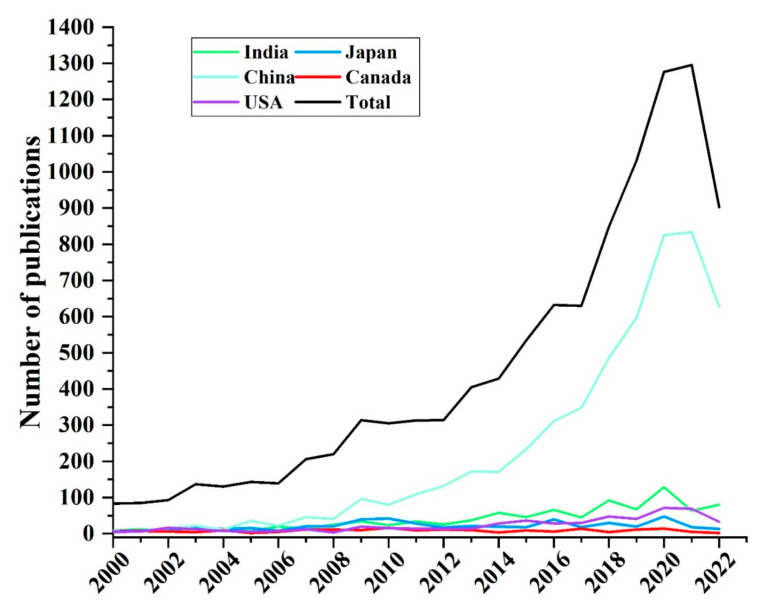
Annual trends in literature on cadmium pollution in rice and wheat cropping system from 2000 to 2022.

The main research fields that explored Cd pollution in the rice and wheat cropping system included environmental science, soil science, agricultural science, plant science, and environmental toxicology. The results showed that the most studied discipline was environmental science, followed by environmental toxicology research. Papers that reported findings on Cd pollution in rice and wheat cropping system were mainly research articles. Most articles published between 2000 and 2022 were mainly from the Environmental Science, Soil Science, Environmental Toxicology, and Plant Science fields, indicating that studies on Cd contamination in rice and wheat cropping system have been explored under these four categories for several years. Articles published in the top 10 most common journals are presented in Table 3. The Journal of Environmental Science and Pollution Research, Ecotoxicology and Environmental Safety and Science of the Total Environment had the highest number of published articles, with three hundred or more articles in each journal. The Chinese Academy of Sciences (China) had the highest number of published articles, followed by Nanjing Agricultural University (China) and Zhejiang University (China) (Figure 5). Internationally, the top five countries in terms of the number of articles on Cd contamination were China, Japan, India, the United States, and Canada. The distribution of the number of publications between 2000 and 2022 is shown in Figure 6. A total of 10,465 publications have been published since 2000. The number of articles on Cd contamination in rice and wheat cropping system published increased between 2002 and 2022. A significant increase in the number of articles in this field has been observed since 2016. A total of 1295 articles were published in 2021, which is 15.6 times higher than the number of articles published in 2000. At the current rate of growth in paper publications, the number in 2022 will be higher than in any other year. The number of articles shared among China, Japan, India, the US, and Canada indicates that these countries have collaborated on research on Cd contamination in rice and wheat cropping systems. The collaboration comprised joint studies by researchers from different countries (Supplementary Materials).

### 5.2. Research Prospects

**Figure 7:**
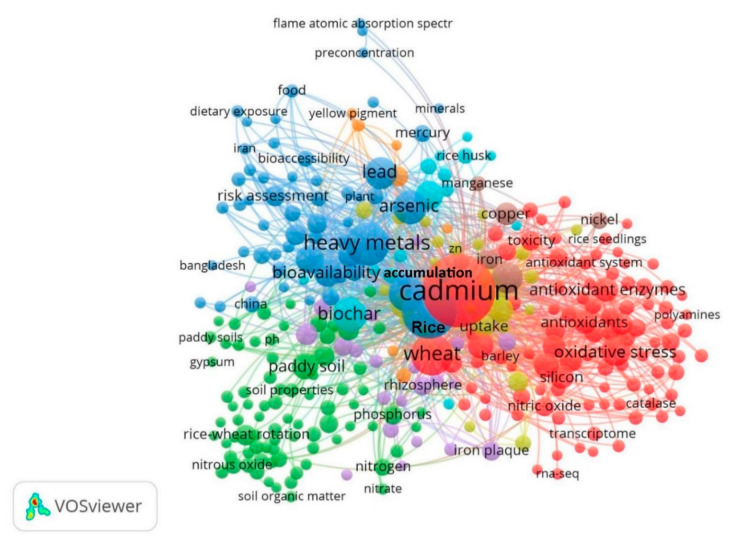
Research hot keyword analysis in rice and wheat cropping system of cadmium pollution.

In addition to keywords such as “Bioavailability”, “Heavy metals”, “Wheat”, “Arsenic”, and “Biochar”, “Cadmium”, “Lead”, “Mercury”, and “Copper” were explored as they are pollutants that significantly affect wheat and rice production (Figure 7). The results showed that Cd had the highest research frequency and the strongest correlation. Cd is highly water-soluble, has high bioavailability, and is readily absorbed by plants. Therefore, it easily enters the food chain, thereby threatening human health. The current research hotspots and progress of Cd pollution in the rice and wheat cropping system were summarized through a blue, red, and green cluster analysis. The keyword “Bioavailability” in the blue cluster indicated that research on heavy metal pollution in rice and wheat cropping systems in recent years mainly focused on the bioavailability characteristic. The keyword “Risk assessment” indicated that the research mainly focused on the potential ecological risks of heavy metals to conduct a hazard assessment of heavy metals in polluted areas. Wheat can accumulate high amounts of Cd mainly through the roots compared with other grains. The Cd is transferred to the aerial parts of the wheat plant and finally accumulates in the wheat [178,179]. Therefore, wheat products are major sources of Cd intake in humans. Keywords such as “Rice field soil”, “Rice wheat”, “Soil properties”, and “Root system” in the red cluster indicated that the recent research mainly focused on the evaluation of changes in paddy soil properties to determine Cd levels in soil roots under the rice and wheat cropping system. Cd has a higher toxicity to wheat than other heavy metals such as Cr [35]. Cd toxicity reduces uptake and transport of essential elements in plants, including wheat [36]. Cd toxicity significantly affects wheat root growth and morphology. Keywords such as “Wheat”, “Antioxidative enzymes”, “Toxicity”, and “Oxidative stress” in the red cluster indicated that recent studies mainly explored the role of the antioxidant enzyme system of wheat in alleviating toxicity of heavy metals in vivo. Cd toxicity promotes several physiological changes in photosynthesis, the reduction of sugar and soluble proteins, and the modulation of antioxidant enzyme activities in shoots [37,38,180]. In addition, Cd toxicity increases reactive oxygen species (ROS) production, leading to oxidative stress in plants [39,181,182,183]. Oxidative stress causes a reduction in plant growth, biomass, and yield [184]. The articles included in this review indicated that the overproduction of reactive oxygen species (ROS) is the main mechanism for Cd toxicity in wheat and other plants [38,185]. High Cd levels increase the malondialdehyde (MDA) content in the shoots and roots of wheat [19]. Notably, plant responses to Cd toxicity vary among wheat cultivars. For instance, the durum wheat variety accumulated higher Cd concentrations than the bread wheat variety [180,186,187,188,189]. The rice and wheat cropping system is a common crop planting practice used in the Yangtze River Delta region of China. Most studies on rice and wheat cropping systems included in this review were mainly on changes in nutrient budgets [67], carbon sinks [190], and other agricultural production parameters. Therefore, further studies on the accumulation and pollution of heavy metals under the crop rotation system should be conducted. The results reported in this study provide a basis for the formulation and application of agronomic measures for rice and wheat cropping systems to reduce heavy metal pollution.

## 6. Conclusions

The present situation of Cd pollution in rice- (*Oryza sativa* L.) and wheat- (*Triticum aestivum* L.) growing areas in China was reviewed. The increasing concentration of Cd in the rice and wheat growing systems in the Yangtze River Basin is of great concern, especially in Hunan Province. In addition, the health exposure risks of Cd pollution in rice-wheat planting systems and current research hotspots were reviewed. The results showed that soil physical and chemical properties, soil microorganisms, and rice/wheat varieties greatly influenced the accumulation of Cd in rice and wheat grains, and regulating these factors could reduce the accumulation of Cd in rice and wheat growing systems. Consumption of Cd-contaminated foods is the main route of human exposure to Cd, so it is imperative to develop strategies to reduce Cd contamination in rice and wheat growing systems. Reducing the transfer of Cd at the source by reducing human activity could significantly reduce the input of Cd and other heavy metals into farmland. The use of agronomic practices, remediation techniques, and planting of low-accumulation varieties can reduce the total contents and bioavailable of Cd. In addition, the mechanism of Cd uptake and transport from the soil by rice and wheat is regulated by a variety of factors. New environmental monitoring methods (such as remote sensing technology) should be adopted to realize large-scale and real-time monitoring of heavy metal pollution in the future. In addition, the accumulation pathway and redistribution process of Cd in food should be further studied to reduce the Cd content in crops.

## Figures and Tables

**Table 1:** The cadmium pollution of rice and wheat cropping systems in eleven provinces in China.

Province	Mean/Rice_cd_ (mg kg^−1^)	Range (mg kg^−1^)	Mean/Wheat_cd_ (mg kg^−1^)	Range (mg kg^−1^)	Reference
Jiangsu	0.0532 ± 0.0204	0.0040~0.4190	0.1050 ± 0.0320	0.0150~0.8700	[55,56,57,58,59,60,61,62,63,64,65,66,67]
Zhejiang	0.1216 ± 0.0378	0.0200~0.3400	0.9010 ± 0.3361	0.0233~1.30	[68,69,70,71,72]
Anhui	0.0860	0.0860	0.0358 ± 0.0250	0.0358~0.0102	[62,73,74]
Hunan	0.4322 ± 0.1513	0.0420~1.415	0.8720 ± 0.3185	0.0400~1.57	[62,71,74,75,76,77,78]
Jiangxi	0.0605 ± 0.0185	0.0420~0.0790	—	—	[79,80]
Sichuan	0.4403 ± 0.2123	0.1100~1.4500	0.3448 ± 0.1896	0.0400~0.4400	[5,68,81,82,83,84,85]
Hubei	0.0460	0.0460	0.0533 ± 0.0233	0.0300~0.100	[61,86,87]
Chongqing	0.0462 ± 0.0143	0.0220~0.0870	0.0080	0.0080	[88,89]
Shanghai	0.0448 ± 0.0111	0.0190~0.0820	—	—	[90]
Henan	0.0046	0.0046	0.0482 ± 0.0174	0.0100~0.6300	[62,73,91,92,93]
Shangdong	—	—	0.0435 ± 0.0061	0.0100~0.0920	[61,94]

**Table 3:** Analysis of the publication sources for studies on cadmium pollution in rice and wheat cropping system from 2000 to 2022.

Rank	Publication Sources	Number of Literature
1	Environmental Science and Pollution Research	425
2	Ecotoxicology and Environmental Safety	339
3	Science of the Total Environment	297
4	Chemosphere	263
5	Environmental Pollution	238
6	Journal of Hazardous Materials	212
7	Plant and Soil	169
8	Communications in Soil Science and Plant Analysis	101
9	Fields Crops Research	93
10	Scientific Reports	87

## Data Availability

The data presented in this study are available on request from the corresponding author.

## Supplementary Materials

The following supporting information can be downloaded at: https://www.mdpi.com/article/10.3390/toxics10120794/s1.
